# Innovative Use of Kiwano Cloud Point Extract in Bioactive Nanoemulsion Development

**DOI:** 10.3390/foods15111909

**Published:** 2026-05-28

**Authors:** Teodora Marić, Nenad Ćetković, Tamara Erceg, Ana Salević, Bojana Balanč, Miroslav Hadnađev, Gordana Ćetković, Vanja Travičić

**Affiliations:** 1 Faculty of Technology Novi Sad, University of Novi Sad, Bulevar cara Lazara 1, 21000 Novi Sad, Serbia; tamara.erceg@uns.ac.rs (T.E.); gcetkovic@uns.ac.rs (G.Ć.); vanjaseregelj@tf.uns.ac.rs (V.T.); 2 Medical Faculty, University of Novi Sad, Hajduk Veljkova 3, 21000 Novi Sad, Serbia; nenad.cetkovic@mf.uns.ac.rs; 3 Department of Food Technology and Biochemistry, Faculty of Agriculture, University of Belgrade, Nemanjina 6, 11080 Belgrade, Serbia; ana.salevic@agrif.bg.ac.rs; 4 Innovation Centre of the Faculty of Technology and Metallurgy, University of Belgrade, Karnegijeva 4, 11000 Belgrade, Serbia; bisailovic@tmf.bg.ac.rs; 5 Institute of Food Technology in Novi Sad, University of Novi Sad, Bulevar cara Lazara 1, 21000 Novi Sad, Serbia; miroslav.hadnadjev@fins.uns.ac.rs

**Keywords:** kiwano peel, cloud point extraction, pectin, CMC, closed-loop process, bioactive nanoemulsion, edible coatings

## Abstract

Kiwano (*Cucumis metuliferus*) peel, although rich in carotenoids and polyphenols with notable antioxidant capacity, is still an underused resource. This study explored its valorization through cloud point extraction (CPE) and its direct integration into nanoemulsion systems. The innovative aspect lies in reusing the CPE as a stabilizing agent, creating a closed process that efficiently incorporates bioactive compounds. In the first phase, oil-in-water nanoemulsions were prepared with sunflower oil, Tween 80, and different polysaccharides (pectin, carboxymethyl cellulose—CMC, and their blends). Their stability, droplet size, surface charge, rheological, mechanical, and barrier properties were thoroughly assessed. CMC-based formulation displayed the most favorable characteristics, particularly strong stability and tensile strength, and was selected as the optimal system. In the second phase, kiwano peel extract was incorporated at three concentrations (5, 10, and 15 wt%). While extract addition lowered tensile strength, it improved elongation at break, suggesting a plasticizing effect. Moreover, extract-loaded emulsions exhibited smaller droplets, high stability, and significantly enhanced antioxidant activity compared to unloaded systems. These results demonstrate that kiwano peel can be sustainably valorized through CPE and integrated into nanoemulsions, offering promising bioactive formulations for future applications in food science.

## 1. Introduction

In recent years, the growing demand for plant-derived functional ingredients has fostered significant advancements in the development of green extraction techniques and the application of innovative technologies to create bioactive formulations [1]. A key aspect of this shift is the need for sustainable approaches that minimize the reliance on synthetic chemicals while maximizing the bioavailability and stability of bioactive compounds [2]. Among plants, kiwano has gained attention for its unique nutritional quality, health benefits, and novel applications [3]. Kiwano, commonly known as the horned melon, African horned cucumber, spiked melon, or jelly melon, is a tropical fruit rich in antioxidants, vitamins, and bioactive compounds. These compounds have been linked to a range of beneficial effects, including anti-inflammatory, antimicrobial, and antioxidant properties [4], which makes kiwano a valuable source for potential applications in the food, pharmaceutical, and cosmetic industries. In addition to its edible part, the kiwano peel, often discarded as waste, contains a diverse phytochemical profile [3,5]. Upcycling the kiwano peel reduces waste and, more importantly, enhances its value, supporting a circular approach to fruit processing.

To align with the principles of green chemistry and sustainable processing, cloud point extraction (CPE) stands out as a promising technique for recovering phenols and carotenoids from kiwano peel [6,7]. CPE offers an environmentally friendly and cost-effective alternative to conventional methods, using surfactants to separate bioactives with different polarities at specific temperatures. This technique is especially significant for carotenoid compounds since conventional aqueous extraction methods are inefficient for their recovery [2,6]. When a specific temperature is achieved, dehydration of the hydrophilic portion of the micelle occurs, leading to the formation of clouding. The structure and concentration of the surfactant determine the cloud point temperature value [8]. Further, the surfactants used in CPE can be selected based on their ability to target specific bioactive compounds, thereby further enhancing the efficiency of the extraction process [2,6]. Moreover, synergistic surfactant utilization can facilitate both the extraction of bioactive compounds and the CPE process. This closed-loop approach enables the direct reuse of the surfactant-rich phase obtained during cloud point extraction in the nanoemulsion formulation, while maintaining a controlled surfactant concentration. This contributes to improved process efficiency and reduced consumption of auxiliary substances, in line with green chemistry principles and sustainability metric [9,10]. Also, this surfactant’s dual role can be an innovative approach in the preparation of high-value plant extracts. Namely, the selective nature of the CPE technique ensures that the compounds of interest are extracted with high yield, thus facilitating their integration into high-value formulations. Kiwano peel extract obtained by CPE has demonstrated strong antioxidant activity [6], primarily due to its carotenoid and polyphenol content, which plays a crucial role in mitigating oxidative degradation processes that compromise the quality, nutritional value, and shelf life of fruits and vegetables during the postharvest phase [11]. Given these properties, its application in the development of edible coatings appears highly promising, offering an innovative approach to prolonging freshness and enhancing sustainability in food preservation.

Besides the extraction of target bioactive compounds, the demanding challenge is to develop formulations that can effectively deliver them while preserving their stability and bioactivity. A promising solution is the development of bioactive nanoemulsions, which are kinetically stable, submicron-sized dispersions of oil and water stabilized by surfactants [12]. In this regard, integrating the extraction process of active components with the preparation step of the edible coating formulation is crucial. Utilizing CPE for obtaining active compounds and formulating active nanoemulsion coating enables integrative approaches, applying a strategy for improving food safety. To the best of our knowledge, there are no studies concerning the integration of CPE plant extracts into emulsion-based edible coating, making this approach innovative in the field of bioactive formulations for food protection. In this approach, the surfactant used in CPE for the extraction of plant bioactives can be repurposed for stabilizing emulsions, creating a more sustainable and efficient system [6]. This creates a closed-loop system, where the CPE is fully utilized, minimizing waste and maximizing the extraction yield. By using the same surfactant for both extraction and formulation, the process becomes more environmentally friendly and cost-effective, minimizing the need for additional chemicals. Nanoemulsions offer an effective delivery system for both hydrophilic and lipophilic bioactive compounds. Due to their specific properties, nanoemulsions, which are used as spray coatings, bring numerous advantages. The small droplets (typically with an average diameter in the 50–500 nm range) enable enhanced kinetic and thermodynamic stability, better adhesion and penetration to different surfaces, and more effective delivery of active ingredients. This results in coatings that are extremely uniform, long-lasting, and more functional, with improved barrier properties or the possibility of controlled release of certain substances. Precisely because of these properties, emulsions are a versatile and useful tool for application in active food packaging [13]. In the development of active packaging and increasing its scalability, the aim is to reduce the number of steps, which would ensure savings in time and resources, and make the process more energy efficient. Different researchers have prepared oil-in-water nanoemulsions using essential oils as the dispersive phase (cinnamon, orange peels, marjoram, oregano, thyme, peppermint, lemongrass essential oil, cardamom, etc.), which were prepared with the addition of polysaccharides such as chitosan, pullulan, pectin, and carboxymethyl cellulose (CMC) [14,15,16,17,18,19,20]. The choice of the coatings ingredients depends on their functional characteristics and their limitations. The various essential oils were used for the production of coatings due to their strong antioxidant and antimicrobial activities, as well as for providing excellent barrier properties against the loss of humidity; however, their use brings unpleasant smell and taste. On the other hand, proteins and polysaccharides can form strong interactions between molecules, resulting in good mechanical and gas barrier (O2 and CO2) properties, but their main limitation is their hydrophilic nature, which leads to poor barrier against the loss of humidity. Because no single coating solution has been identified as the most adequate, researchers are investigating a range of ingredient combinations [21].

In the preparation of nanoemulsion edible coatings with lyophilic compounds dissolved in carrier oils, different polysaccharides (pectin, alginates, starch, carrageenan, gums, etc.) and proteins (soy, whey proteins, etc.) have been applied for providing structural support [22]. However, despite an extensive review of the literature, the interfacial interactions between CPE- derived surfactants and PEC/CMC-based matrices in a sunflower oil system remain rarely understood, particularly regarding their impact on bioactive release kinetics. Although numerous coatings have been developed, economic viability and availability of raw materials remain significant challenges. In order to solve this problem, our work investigates the potential of using sunflower oil in combination with cheap biopolymers. Our primary hypothesis is that the combination of pectin and carboxymethyl cellulose, which is significantly more cost-effective than other biopolymers, will enable the formation of stable emulsions and effective, continuous coatings on the fruit surface. In the first phase of this study, the objective was to develop a stable, unloaded emulsion system. This involved screening two widely available biopolymers, pectin (PEC) and carboxymethyl cellulose (CMC), as well as their combinations. The resulting emulsions were fully characterized in terms of their stability, droplet size, and rheological properties. Additionally, films obtained by solution casting were investigated for their barrier and mechanical properties. Through investigation of rheological behavior, barrier and mechanical properties, this study offers a valuable insight into structural integrity and functional performances. Further, kiwano CPE was then incorporated into the optimized nanoemulsion formulation at three different concentrations. These resulting emulsions were characterized using the same methods, with the addition of antioxidant activity assays to evaluate the biofunctional potential of the extract-enriched systems.

## 2. Materials and Methods

### 2.1. Materials

Horned melon fruits were purchased from an organic farm situated near Novi Sad, Republic of Serbia. The horned melons’ peels were manually detached in sterile lab conditions, following the freeze-drying process in a Martin Crist Alpha 2–4 (Osterode, Germany). The collected freeze-dried horned melon peel sample was pulverized and stored until further use. Tween 80 and carboxymethyl cellulose, sodium salt, Mw = 90,000, DS = 0.7, was obtained from Thermo Scientific (Waltham, MA, USA). Pectin from citrus peel, galacturonic acid ≥ 74.0% dried basis, was obtained from Sigma Aldrich (St. Louis, MO, USA). All other chemicals were analytical grade.

### 2.2. Preparation of Unloaded Nanoemulsions

**Figure 1:**
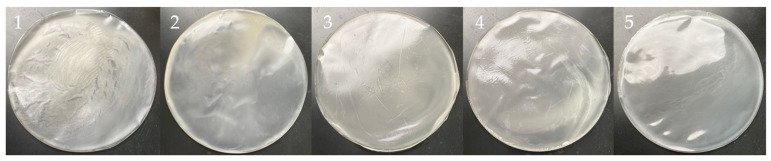
The appearance of unloaded nanoemulsions-films: (**1**) PEC100/CMC0; (**2**) PEC75/CMC25; (**3**) PEC50/CMC50; (**4**) PEC25/CMC75; and (**5**) PEC0/CMC100.

The preparation of unloaded nanoemulsions in this study involved a two-step process. First, emulsions and biopolymer solutions were prepared separately. The emulsions were created by mixing sunflower oil, water, and surfactant in a ratio of 1:96:3 *w*/*w*. These components were mixed using an Ultra-Turrax at a speed of 11,000 rpm for 1 min to obtain a coarse emulsion. The biopolymer solutions were prepared by dissolving pectin (PEC) and carboxymethyl cellulose (CMC) in distilled water at 60 °C for 2 h using a magnetic stirrer. The ratios of PEC and CMC varied as shown in Table 1, while the total concentration of biopolymers in the nanoemulsions was maintained at 2% (*w*/*w*). Glycerol was added to the biopolymer solution at a concentration of 30% (*w*/*w*) relative to the final biopolymer concentration. The second step included mixing the prepared emulsion with biopolymer solution in a ratio of 1:1. The final nanoemulsion contained 2% (*w*/*w*) of biopolymer, 1.5% (*w*/*w*) of surfactant, 0.5% (*w*/*w*) of sunflower oil, and 0.6% (*w*/*w*) of glycerol. The obtained formulation was mixed by Ultra-Turrax (Ultra-Turrax^®^ T25, Ika-Labortechnik, Staufen, Germany) at a speed of 11,000 rpm for 3 min to obtain the coarse nanoemulsion, which was followed by ultrasonication. The operational conditions of a sonotrode (UP400St ultrasonic processor, Hielscher, Germany) were 10 min at an amplitude of 60%, where the power of the sonification was in the range of 104 to 119 W. The ultrasonication parameters (60% amplitude for 10 min) were determined through preliminary optimization trials aimed at balancing emulsification efficiency with energy conservation. Initial testing indicated that these conditions provided sufficient acoustic energy to achieve a stable droplet-size distribution. While higher amplitudes or longer durations might further reduce droplet size, they were avoided to prevent thermal decomposition of the bioactive compounds and to ensure energy efficiency. Furthermore, the temperature was maintained below 55 °C using an ice bath to preserve the structural integrity of the nanoemulsion components during cavitation. The nanoemulsion system was heated during ultrasonication from 15 °C up to 55 °C. To determine structural, mechanical, and barrier properties, the obtained nanoemulsions were poured into Petri dishes and dried in a laboratory oven at 50 °C for 24 h. Unloaded nanoemulsions-films are presented in Figure 1.

### 2.3. Cloud Point Extraction Procedure

**Figure 2:**
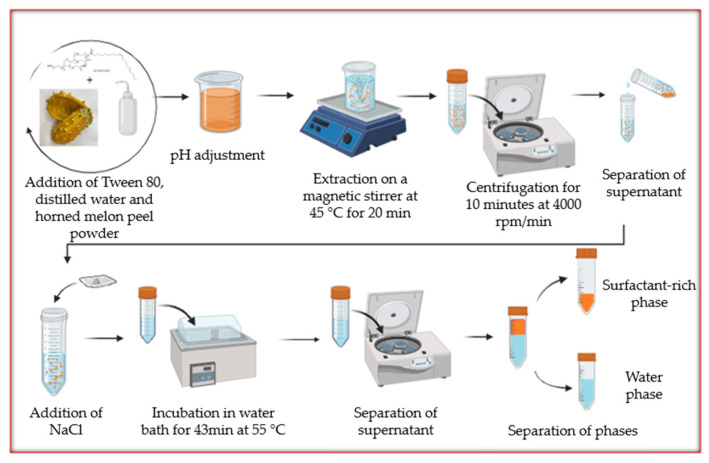
Schematic diagram of cloud point extraction (CPE).

Cloud point extraction (CPE) was performed according to the procedure in Travičić et al. 2024 [6]. Firstly, the horned melon powder was added in a solid-to-liquid ratio of 1:70 (*w*/*v*) along with 10% (*w*/*v*) of Tween 80 to the flask. The pH value of the solution was set at 7.3 and stirred for 20 min on a magnetic stirrer at 45 °C (Magnetic stirrer Hei-PLATE MIX′N′HEAT CORE, Heidolph, Schwabach, Germany). Afterward, the supernatant was collected after centrifugation of the solution for 10 min at 4000 rpm (Z206-A Compact Centrifuge, Hermle AG, Gosheim, Germany). In the obtained supernatant, 18% (*w*/*v*) of NaCl and the mixture were kept in a water bath (HB4000 Digital WaterBath, Heidolph, Schwabach, Germany) at a temperature of 55 °C for 43 min. After incubation, the separation of the surfactant-rich phase was noticeable. The sample was centrifuged at 4000 rpm for 10 min to achieve profound phase separation, and the bottom aqueous phase was removed using a pipette. The cloud point extraction (CPE) procedure is presented in Figure 2.

### 2.4. Preparation of Extract-Loaded Nanoemulsions

**Figure 3:**
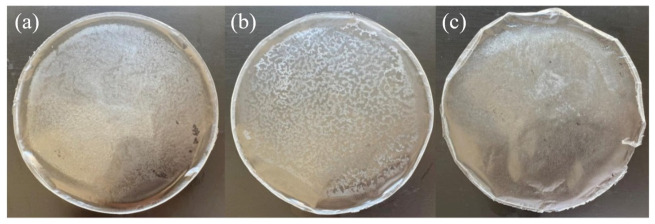
The appearance of loaded nanoemulsions-films: (**a**) 5%CPE, (**b**) 10%CPE and (**c**) 15%CPE.

The loaded nanoemulsions were prepared as described in section Preparation of unloaded nanoemulsions with slight modification. The change was made to the emulsion preparation. Namely, the CPE contains Tween 80 along with bioactive compounds. Different amounts of this phase (5, 10, and 15 wt%) were incorporated into the nanoemulsions. The total surfactant concentration in all formulations was adjusted to 3 wt% by correcting the amount of additional Tween 80, considering the surfactant contribution from the CPE-derived phase. The blank sample was prepared using 3% of Tween 80. Testing the unloaded nanoemulsions highlighted the use of formulation 5 (PEC0/CMC100), prepared with CPE concentration variation (5, 10, and 15 wt% per biopolymers mass). The loaded formulations are presented in Figure 3, where their visual appearance demonstrates physical stability, which is further evaluated through analyses of rheological behavior, barrier, and mechanical properties, as well as particle size, zeta potential, PDI, mobility, and conductivity.

### 2.5. Determination of Zeta Potential (ζ), Polydispersity Index, Mobility, Conductivity, and Size Distribution

Particle size, zeta potential, PDI, mobility, and conductivity were monitored using the Malvern Zetasizer Nano ZS (Malvern Instruments, Worcestershire, UK) with a He–Ne laser at 25 ± 0.1 °C. Each nanoemulsion sample was diluted 50x with distilled water. The measurements were repeated three times, and the results were reported as a mean value with standard deviation. The stability of unloaded nanoemulsions was measured on days 1, 7, 14, 21, 30, 45, and 60 during a 60-day storage period at 4 °C [23]. For loaded nanoemulsions, particle size, zeta potential, PDI, mobility, and conductivity were determined on the 1st day. Furthermore, the stability of the loaded nanoemulsions was monitored over 14 days, with droplet size measurements performed on days 1, 3, 7, and 14.

### 2.6. Analysis of Rheological Properties of Nanoemulsions

The rheological behavior of the nanoemulsions, designed to mimic storage conditions, was characterized by measuring their flow properties at a stable temperature of approximately 23 ± 1 °C. To construct flow curves of the film-forming solutions, stress and viscosity values were calculated as a function of shear strain rate, which progressively increased from 1.91 to 100 s^−1^ and subsequently decreased back to 1.91 s^−1^ at a controlled temperature.

### 2.7. Film Thickness Determination

The film thickness was determined at five randomly selected points in each sample via a digital micrometer with an accuracy of 0.001 mm (S00014, Mitutoyo Corporation, Kawasaki, Japan).

### 2.8. Analysis of the Mechanical Properties of the Obtained Films

**Figure 4:**
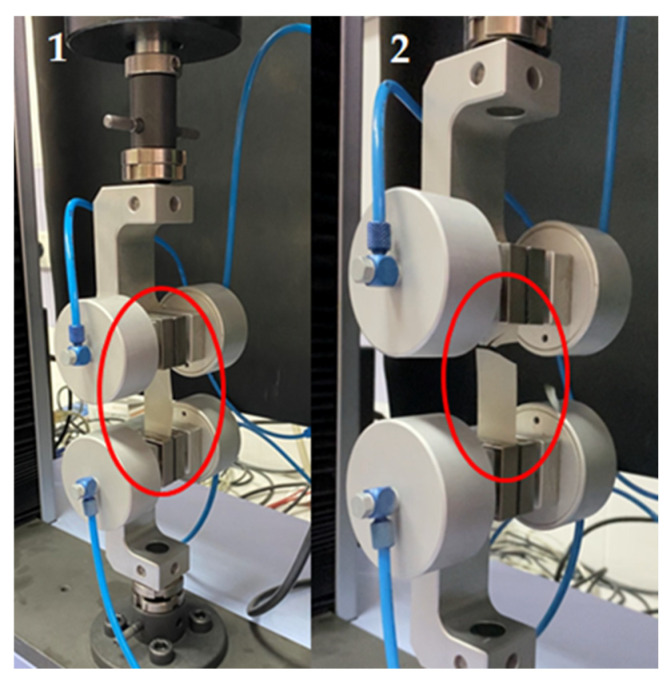
Tensile testing of films using Universal Testing Machine; (1) sample between the grips before testing, and (2) sample after the fracture.

Mechanical properties of films were determined using the Universal Testing Machine Shimadzu AG–X Plus test machine (Shimadzu, Kyoto, Japan, Figure 4). Before testing, samples were shaped into rectangular strips and stretched with a cross-head speed set at 10 mm/min. For each sample, measurements were carried out five times, and the average value of elongation at break (EB) and tensile strength (TS) was calculated.

### 2.9. Water Vapor Permeability (WVP) Test

The method for the determination of water vapor permeability (WVP) is described in the work of Salević et al. 2019 [24]. Briefly, the prepared films were cut into squares and placed in Payne permeability cups and sealed, where there was 5 mL of distilled water, which provided a 100% RH exposure to one side of the films. On the other side, exposure to 0% RH was achieved when cups were put in a desiccator. The cups were left at ambient temperature and weighed regularly until a constant level was obtained. As sample controls, the cups with aluminum foil with water and films without water in the cups were used. Through analyses of weight loss decline in time and multiplying by film thickness, the water vapor permeability rate (WVPR) was calculated. The analysis was done in triplicate.

### 2.10. Antioxidant Activity Analysis

The antioxidant activity of the extract-loaded naonemulsions was evaluated using the three assays. The assays followed a spectrophotometric procedure described by Travičić et al. [6].

DPPH radical scavenging assay: Briefly, 10 µL of the sample was mixed with 250 µL of a methanolic DPPH^•^ solution (0.89 mM) in a microplate well. The mixture was incubated for 50 min in the dark at room temperature, after which absorbance was read at 515 nm. Methanol served as the blank. Results were expressed as µmol Trolox equivalents (TE) per 100 mL of sample.

Reducing power: In each well, 25 µL of sample (or distilled water as blank), 25 µL of sodium phosphate buffer (pH 6.6), and 25 µL of 1% potassium ferricyanide were combined and incubated at 50 °C for 20 min. After cooling, 25 µL of 10% trichloroacetic acid was added, and the mixture was centrifuged at 4000 rpm for 10 min. Subsequently, 50 µL of the supernatant was mixed with 50 µL of distilled water and 10 µL of 0.1% ferric chloride. The absorbance was measured immediately at 700 nm. Results were expressed as µmol Trolox equivalents (TE) per 100 mL of sample.

ABTS radical scavenging activity: Activated ABTS^+•^ solution (250 µL, prepared using MnO2) was added to 2 µL of the sample and incubated at 25 °C for 35 min. The absorbance was measured at 414 nm before and after incubation. Distilled water served as the blank. Results were expressed as µmol Trolox equivalents (TE) per 100 mL of sample.

### 2.11. Statistical Analysis

Statistical analysis was performed using Microsoft Excel software (Version 2022). Data are presented as mean values ± standard deviation. The significance of differences between the means was determined using One-way Analysis of Variance (ANOVA), followed by a pair-wise comparison using Student’s *t*-test. Statistically significant differences were defined at a confidence level of *p* < 0.05. In all tables, different superscripts (e.g., a, b, c) indicate groups that differ significantly, whereas shared superscripts indicate no statistically significant difference (*p* > 0.05).

## 3. Results and Discussion

### 3.1. Results of Particle Size, PDI, and Zeta Potential Analysis

The droplet size, polydispersity index (PDI), and zeta potential of the unloaded nanoemulsions were evaluated on days 1, 7, 14, 21, 30, and 60 during the 60-day storage. Results are given in Table S1 in the Supplementary Material. On day 1, the droplets were the smallest in formulation 5, reaching the value of 218.2 nm, which was also confirmed by visual inspection. During monitoring, the size of the droplets changed, indicating their coalescence and eventual phase separation. Fluctuations in size were especially noticeable during the first 14 days. Namely, some changes in the average droplet size are present in all samples during the reported period, suggesting ongoing physical processes in the samples. The average size of emulsion droplets decreased over time, indicating aggregation or coalescence processes governed by different flocculation mechanisms. This claim can also be supported by the fact that the results of rheological tests suggested Newtonian flow. Namely, Otsubo et al. reported that suspensions flocculated via reversible polymer bridging exhibit Newtonian behavior at very low shear rates due to continuous bridge rearrangements [25]. On the other hand, Bai et al. (2017) suggested that emulsions can remain stable against depletion flocculation at relatively low polysaccharide concentrations; however, aggregation may occur once a critical polysaccharide concentration is exceeded [26]. The rapid phase separation and increase in particle size in the emulsions with both pectin and CMC can also be explained by depletion flocculation induced by non-adsorbed anionic polysaccharides in the continuous phase [27]. After day 30, formulations 1, 3, and 5 had completely separated. Formulations 1 and 5 were also separated, but not completely, as evidenced by the fact that smaller droplets were still present even at 45 and 60 days. This probably means that part of the oil phase remained emulsified. Sample 5, which was our focus in further experiments, displayed a slight decrease in particle size, together with similar values of PDI and a slight decrease in zeta potential over the same time range. This behavior of the emulsion is more consistent with a rearrangement or compaction of the interfacial biopolymer/surfactant layer and changes in the location of charged components, which can also alter the surface potential of the particles [28].

When compared to similar systems utilizing a combination of Tremella polysaccharide and pectin, the prepared emulsions exhibit significantly lower droplet size values. This disparity underscores the critical influence of the polysaccharide architecture and the resulting stabilization mechanism. While the Tremella-pectin system relies on a pseudoplastic (shear-thinning) profile—typically associated with a high-viscosity continuous phase that physically “traps” droplets—the prepared system’s Newtonian behavior indicates a more efficient, interfacial-dominant stabilization. The presence of a non-ionic surfactant in this formulation rapidly lowers the interfacial tension during emulsification, facilitating the formation of smaller droplets. The subsequent interaction between the surfactant and the polysaccharide phases is fundamental to maintaining this morphology. At the oil-water interface, the linear backbone of CMC aligns with the polyoxyethylene (POE) chains of Tween 80, effectively “wrapping” the droplets in a dense, hydrated shell. This assembly provides massive steric hindrance, creating a robust physical barrier against coalescence. Simultaneously, at neutral or slightly acidic pH, the deprotonated carboxyl groups (-COO^−^) of the CMC chains impart a strong negative zeta potential, ensuring long-term stability through electrostatic repulsion. In contrast to Tremella-Pectin complexes, which often form bulky, shear-sensitive 3D networks in the continuous phase, the specific ratio of CMC in this formulation appears to suppress the formation of large-scale pectin entanglements. Instead, the pectin works in tandem with CMC to form a thin, highly structured interfacial layer rather than a bulk-phase matrix. Because these biopolymers exist as discrete, non-entangled chains in the aqueous phase, the system avoids the formation of a shear-sensitive “mesh.” This preserves the Newtonian flow profile [29].

The zeta potential represents the surface charge of the colloidal particles (ζ), indicating the interactions between the biopolymers within the particles and their tendency to aggregate. Zeta potential values greater than +30 mV or lower than −30 mV is considered to reflect stable systems [29]. Consistent with the stability test results, formulations 1 and 5 exhibited the greatest absolute zeta potential values, indicating higher stability. Bai et al. (2017) suggested that emulsions can remain stable against depletion flocculation at relatively low polysaccharide concentrations; however, aggregation may occur once a critical polysaccharide concentration is exceeded [26]. The rapid phase separation and increase in particle size in the emulsions with both pectin and CMC are consistent with depletion flocculation induced by non-adsorbed anionic polysaccharides in the continuous phase. Similarly, Mirhosseini et al. reported that high concentrations of pectin and CMC can lead to undesirable increases in droplet size and physical instability [27]. The reduction in zeta potential value (compared to emulsions with only one polysaccharide) further supports this explanation and it is likely associated with a shift in the slipping plane due to the formation of a thicker interfacial layer. Namely, the anion-anion repulsion between pectin and CMC increases the hydrodynamic thickness of the droplets and simultaneously shifts the slipping plane further from the surface, leading to the observed reduction in zeta potential [30,31]. The absolute zeta potential values also showed fluctuations, correlating with observed changes in droplet size and PDI. Extract-loaded nanoemulsions exhibit lower droplet sizes by 29.2, 104.3, and 6.2% for nanoemulsion samples with 5, 10, and 15% extract, respectively, as measured on the first day. Based on the results, the extract also stabilized the emulsion due to the presence of the surfactant used in the CPE. PDI values are beyond the values of neat nanoemulsions 5, and zeta potential values are similar to the values of the unloaded nanoemulsion recorded for the first day (Table S2, Supplementary Material). Evaluation of the droplet size of loaded nanoemulsions monitored for 14 days provided insight into the behavior of the prepared systems (Figure S1, Supplementary Material). The blank sample, neat nanoemulsions 5, remained relatively stable during the 14 days, where the droplet size varied from 88.22 to 89.56 nm. This system did not undergo major changes. On the other hand, the droplet size of nanoemulsion containing 5% extract gradually increased over time from 93.92 nm on day 1 to 99.89 nm on day 14. Nanoemulsion containing 10% extract showed the highest values of droplet size, ranging from 111.13 nm on day 1 to 135.56 nm on day 14, while nanoemulsion with 15% remained close to the values of blank and 5% nanoemulsion, where droplet size fluctuated from 96.05 to 101.94 nm.

### 3.2. Results of Rheological Properties Analysis

**Figure 5:**
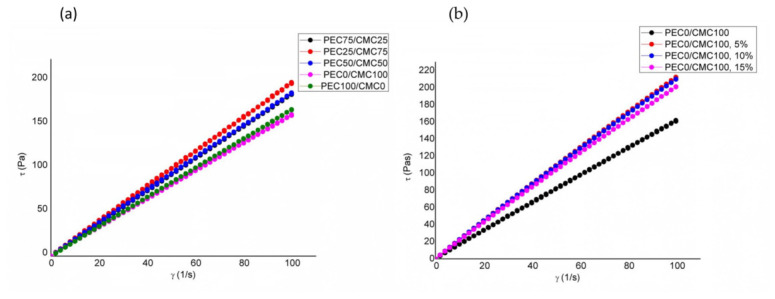
The relationship between shear stress and shear rate for (**a**) unloaded nanoemulsions and (**b**) nanoemulsions with different amounts of CPE: 5, 10, and 15 wt%.

Unloaded emulsions exhibited Newtonian-like behavior, with the highest viscosity observed for the PEC25/CMC75 sample, likely resulting from biopolymer interactions, while the PEC0/CMC100 and PEC100/CMC0 samples displayed the lowest viscosity (Figure 5). The higher viscosity observed for the PEC25/CMC75 system can be attributed to stronger intermolecular interactions between the two biopolymers. CMC is a linear polysaccharide with carboxyl (–COOH/–COO^−^) groups and numerous hydroxyl groups (–OH), whereas PEC has a branched molecular structure with methyl ester groups (-COOCH_3_), numerous hydroxyl and carboxyl functional groups [32]. In the PEC25/CMC75 formulation, the higher proportion of CMC provides extended linear chains capable of limited intermolecular overlap and transient physical interactions, while the branched pectin molecules may act as localized junction points. This structural arrangement facilitates additional hydrogen bonding and short-range chain associations, resulting in a weakly structured continuous phase. Consequently, the mobility of polymer chains and dispersed droplets becomes partially restricted, increasing resistance to flow and leading to higher viscosity values. Despite these interactions, the emulsions still exhibited Newtonian flow behavior, meaning that the viscosity remained independent of the applied shear rate within the investigated range. This indicates that PEC–CMC interactions primarily increase the viscosity of the continuous phase without forming a continuous, percolated polymer network capable of inducing shear-dependent behavior. In contrast, systems containing only one biopolymer (PEC100/CMC0 or PEC0/CMC100) lack these complementary interactions, resulting in weaker intermolecular association and lower viscosity, while still maintaining Newtonian rheological behavior. The addition of extracts increases the viscosity of emulsions. Nanoemulsions with the extract have similar viscosity values. However, nanoemulsions containing 15% extract had lower viscosity compared to those containing 5wt% and 10wt% extract.

In contrast, nanoemulsion systems exhibiting shear-thinning (pseudoplastic) behavior typically have PDI values in the range of 0.1–0.4, which correspond to a narrow and more uniform droplet size distribution [33]. More homogeneous droplet populations facilitate stronger interactions and structural organization within the dispersion, often resulting in non-Newtonian rheological behavior. After the addition of the extract, both particle size and PDI decreased, indicating improved droplet stabilization and a narrower size distribution. This effect is likely related to the higher amount of surfactant originating from the first step (CPE process), which enhances interfacial stabilization and promotes the formation of smaller droplets. Despite the reduction in particle size and PDI, the emulsions still exhibited Newtonian flow behavior. This can be explained by the very high water content of the system, where water represents the dominant continuous phase. Under these highly dilute conditions, droplet–droplet interactions remain limited, and the rheological behavior is primarily governed by the viscosity of the aqueous phase. As a result, the emulsions show constant viscosity independent of shear rate. Therefore, although localized polymer–polymer interactions and improved droplet stabilization are present, the overall low polymer concentration and high dilution prevent the formation of a continuous droplet or polymer network, resulting in the observed Newtonian rheological behavior.

### 3.3. Results of Mechanical Properties Analysis

CMC-rich samples (carboxymethyl cellulose) in their composition—specifically samples 3, 4, and 5—exhibit the greatest tensile strength (TS). The pectin-only sample has the lowest TS value. Higher TS of CMC films can be attributed to the linear structure and the degrees of substitution of carboxyl methyl groups on the cellulose hydroxyls, and thus to crystallinity. The detailed results of the mechanical properties of the prepared loaded and unloaded nanoemulsions are shown in Table 2. Namely, it is known that CMC can form strong films [34,35], while pectin films are more challenging due to the lack of good mechanical properties [36]. The observed decrease in TS upon adding the extract can be due to weakening of intramolecular interactions among polymeric chains caused by the presence of polyphenols from the extract. The extract may exhibit a plasticizing effect leading to decreased rigidity and increased flexibility of the films [37,38]. Sample 5, which is based on neat CMC, also has the highest value for elongation at break (EB), at 10.17%. The addition of the extract leads to a significant decrease in TS, with values dropping by a factor of 3.16 to 5.81. In comparison to the mechanical properties of films obtained from pectin-based nanoemulsion with essential oil as dispersive phase, where the highest obtained TS was 3.95 MPa, which is approximately 6 times lower than the results of this study [16]. An increase in EB values accompanies this reduction in tensile strength. The greatest EB value is observed in the film with the lowest amount of added extract (5 wt%), likely because the extract, in this specific concentration, acts as a plasticizer, enhancing the material’s flexibility and stretch ability. The addition of the CPE reduced TS and increased EB of the films, demonstrating the plasticizing effect of CPE. Namely, as reported by Kokoszka et al., plasticizers alter the three-dimensional structure of polymeric systems, reducing intermolecular forces, increasing free volume, and enhancing chain mobility [39]. These structural modifications result in changes to the functional properties of films, such as increased flexibility, along with reduced rigidity and cohesion. Similar findings were also reported by Otoni et al., who added papaya puree to pectin films [40]. In the study of Saidi et al., testing the different polyphenolics revealed that catechin formed more hydroxyl bonds between this polyphenol and the matrix, leading to an increase in TS and EB [41]. While in the study of Akl et al., the phenolic compounds were extracted from sesame seed meal and incorporated into carboxymethyl cellulose films [42]. They discovered that tensile strength slightly increased by the addition of 0.08% extracted phenolic compounds, while the addition of higher concentrations led to a decrease in TS. The same trend was observed for the elongation break.

### 3.4. Results of WVP Analysis

The water vapor permeability (WVP) values of the unloaded films are presented in Table 3. Overall, films derived from unloaded nanoemulsions exhibited comparable barrier properties against water vapor, although differences were observed depending on the PEC/CMC ratio and film thickness. The highest WVP value was observed for sample 1 (PEC100/CMC0), which contains only PEC, despite having the greatest film thickness. A relatively high WVP was also recorded for the PEC75/CMC25 sample, although its film thickness was more than two times lower. This indicates that the barrier performance is not governed solely by thickness but also by the molecular structure and interactions of the biopolymers within the film matrix. The film containing equal proportions of PEC and CMC (sample 3) exhibited the lowest WVP value, indicating the best barrier properties against water vapor. This behavior can be attributed to hydrogen interactions between PEC and CMC chains, which likely promote better packing and stacking within the polymer network, resulting in a more compact structure. This arrangement increases the tortuosity of the diffusion pathways for water molecules, forcing them to navigate around multiple polymer chains and junctions rather than moving in straight paths. The resulting complex effectively slows down water vapor transport, which is reflected in the lower water vapor permeability (WVP) observed for the PEC50/CMC50 film compared to films with either polymer alone or non-equimolar ratios. Interestingly, the PEC0/CMC100 film, composed entirely of CMC, showed barrier properties similar to those of PEC25/CMC75 (sample 4) and PEC50/CMC50 (sample 3) when films of similar thickness are compared. This behavior may be explained by structural differences between the two biopolymers. Pectin possesses a branched molecular structure, which can hinder the formation of a densely packed matrix and facilitate the formation of microvoids that allow easier water vapor transport [43]. In contrast, CMC has a more linear polymer structure, which promotes closer chain packing and stronger intermolecular interactions, leading to a more compact film structure and improved barrier properties. Therefore, the balance between PEC and CMC composition plays an important role in determining film microstructure and water vapor barrier performance, with intermediate ratios providing the most efficient molecular packing and consequently the lowest water vapor permeability [44]. The addition of the extract increases the permeability of the film, which is a direct result of the extract’s components acting as a plasticizer, increasing the flexibility and decreasing the impermeability of a material. These values are very similar to the WVP values of pectin–based nanoemulsion films prepared with essential oil by Almasi et al. [16].

### 3.5. Results of Antioxidant Activity Analysis

**Figure 6:**
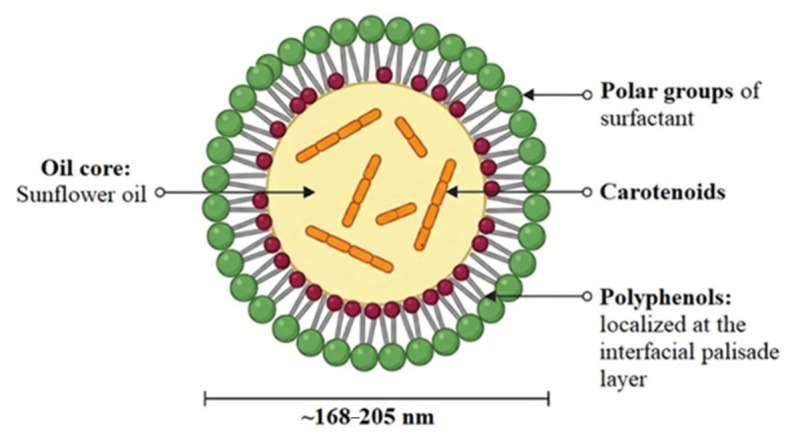
The schematic representation of the extract location in the oil core and surfactant tail region.

In our previous study, the kiwano peel extracts were shown to possess a significant content of total carotenoids and polyphenolic compounds, 13.11 mg β-carotene/100 g and 239.71 mg GAE/100 g, respectively. Further, the antioxidant activity of extracts was evaluated using three tests: DPPH, RP, and ABTS, and the obtained results were 9.05 μmol TE/100 g, 308.63 μmol TE/100 g and 7398.58 μmol TE/100 g, respectively [6]. The antioxidant activity analysis in this study clearly demonstrated that the incorporation of kiwano peel extract enhanced the functional properties of the nanoemulsions in comparison to the blank, which showed no activity (Table 4). The localization of bioactives is determined by their chemical polarity: lipophilic carotenoids (e.g., β-carotene) are sequestered within the hydrophobic oil core [45,46]. At the same time, the more polar polyphenols preferentially localize at the interfacial palisade layer or near the surfactant headgroups (Figure 6). These bioactives do not exhibit significant co-surfactant activity. Instead, increasing the extract concentration above 10 wt% appears to reach a saturation point for the surfactant system. This excess effectively increases the oil-phase volume (due to the lipophilic load) and causes the polyphenols to crowd the polar headgroup region. This crowding interferes with the surfactant-water hydration layer, potentially deteriorating the emulsion stability rather than further reducing interfacial tension. Successfully entrapping the extract’s bioactives is responsible for detecting the antioxidant potential of loaded nanoemulsions. All three assays (DPPH, reducing power, ABTS) exhibited a concentration-dependent increase, confirming that polyphenols and carotenoids from the extract were the main contributors to radical scavenging capacity and reducing power.

DPPH assay revealed values of 0.04, 0.07, 0.19 µmol TE/100 mL for 5%, 10% and 15% CPE loaded nanoemulsion. These results are in line with the lipophilic nature of the DPPH radical, which interacts less efficiently with hydrophilic antioxidants. Higher ABTS radical scavenging activity compared to DPPH was observed across all extract-loaded nanoemulsions, with values increasing proportionally with extract concentration. This response reflects the higher sensitivity of the ABTS assay toward phenolic antioxidants present in hydrophilic and interfacial domains. In addition, reducing power increased proportionally with extract concentration, indicating effective retention of electron-donating compounds within the nanoemulsion matrix. Similar assay-dependent antioxidant profiles have been reported for edible coatings and films incorporating plant-derived actives. Almasi et al. [16] also observed a concentration-dependent increase in DPPH radical scavenging activity in pectin-based films activated with nanoemulsified marjoram essential oil, confirming that antioxidant functionality was preserved after incorporation into the coating matrix. Furthermore, the review article of Eca et al. states that many researchers incorporated natural antioxidants, such as essential oils, plant extracts, and pure compounds (ascorbic acid) into films and edible coatings that expressed higher antioxidant activity than the control film [47]. In the study of Seididamyeh et al., gum Arabic was used with aqueous plant extracts for the preparation of a coating that was applied to fresh-cut red capsicum [48]. The antioxidant activity was evaluated by the DPPH test, and the results revealed that the antioxidant capacity of the edible films was significantly influenced by the linear effects of the extract blend and gum Arabic concentration (DPPH IC50 ranging from 26.36 to 43.73 μg/mL), which is in correlation with this study. The presence of plant extracts with secondary metabolites, such as phenolic compounds, enriches the coating and enhances their antioxidant activity [48].

## 4. Conclusions

This work offers a comprehensive strategy for upcycling kiwano peel, which is a rich source of carotenoids and polyphenols, using green extraction techniques, including cloud point extraction (CPE), in conjunction with the creation of edible coatings based on nanoemulsion. By using the same surfactant for both extraction and emulsion stabilisation, the novel closed-loop technology reduces waste and boosts effectiveness. Rheological studies have demonstrated that the obtained nanoemulsions with and without extract show Newton-like behaviour, which provides a critical mechanical advantage for the development of high-performance sprayable coatings. By maintaining a constant viscosity independent of the shear rate, these formulations ensure uniform droplet atomization and a predictable spray pattern, effectively bridging the gap between liquid rheology and efficient surface deposition. This stability under the high-shear conditions of the spray nozzle eliminates the risk of irregular droplet sizing or sudden pressure fluctuations, which are common challenges with non-Newtonian systems. Most importantly, these emulsions are inherently sprayable, as they do not require additional force or “break-in” shear to initiate flow, allowing for an immediate and consistent discharge through the applicator. Ultimately, the linear relationship between pressure and flow allows for precise calibration and a highly controlled, consistent film thickness. This behavior is superior for applications requiring reliable material distribution, as it minimizes waste and ensures that the active phase is delivered with high precision across the target substrate. All formulations showed satisfactory stability for 21 days, and nanoemulsions prepared only with the addition of one biopolymer—pectin or CMC—maintained stability for 60 days. Samples with a dominant amount of CMC have shown improved ability to withstand stress while being stretched, manifested in high values of TS (above 23 MPa). Addition of extract decreases TS values, but increases the values of EB, acting as a plasticizer in applied concentration (5, 10, and 15wt%). Films based on nanoemulsions with higher amounts of CMC (100, 75, and 50 wt%) possess the best barrier properties, maintaining them after the addition of 5 wt% of extract. The rise in WVP beyond the 5% of extract concentration underscores the importance of identifying an optimal extract concentration that achieves the favorable performances while maintaining barrier efficiency. Antioxidant activity increased proportionally with extract concentration, confirming the functional retention of kiwano peel bioactives within the coating matrix. Based on the results obtained, CMC is a more cost-effective biopolymer that can serve as an efficient substitute for more expensive pectin in the preparation of scalable formulations for postharvest food protection.

While the present study successfully established the fundamental framework for the carrier system and characterized the immediate impact of extract loading on the resulting nanoemulsions, it is recognized that incorporating horned melon extract can significantly influence long-term stability. Therefore, the next phase of this investigation will be devoted to a comprehensive evaluation of the kinetic stability and shelf life of these loaded systems. Future research will focus specifically on monitoring droplet size evolution, phase separation, and the degradation kinetics of the encapsulated compounds under various storage conditions to ensure the practical efficacy and robust performance of the final formulations.

## Figures and Tables

**Table 1:** The content of biopolymers in unloaded nanoemulsions.

Biolymer Ratio (%)	Sample				
	1 PEC100/CMC0	2 PEC75/CMC25	3 PEC50/CMC50	4 PEC25/CMC75	5 PEC0/CMC100
Pectin	100	75	50	25	0
Carboxymethyl cellulose	0	25	50	75	100

**Table 2:** Mechanical properties: tensile strength (TS) and elongation at break (EB) of prepared films.

Sample	TS (MPa)	EB (%)
1	23.58 ± 3.60 ^a^	5.68 ± 2.81 ^ab^
2	23.24 ± 6.35 ^a^	10.17 ± 5.42 ^bc^
3	23.08 ± 5.50 ^a^	4.51 ± 1.03 ^b^
4	7.92 ± 4.74 ^c^	1.29 ± 0.42 ^c^
5	14.47 ± 3.14 ^b^	2.43 ± 1.05 ^a^
5%CPE	7.36 ± 0.87 ^a^	27.78 ± 3.56 ^a^
10%CPE	4. 00 ± 0.71 ^b^	17.70 ± 1.53 ^b^
15%CPE	4.82 ± 1.02 ^b^	22.58 ± 2.18 ^c^

Data are expressed as mean ± standard deviation. Values within the same column followed by the same superscript letter are not significantly different (*p* > 0.05), while different letters indicate statistically significant differences.

**Table 3:** Water vapor permeability (WVP) and thickness of films.

Sample No.	WVP (g/Pa s m)	Thickness (mm)
1	2.59 × 10^−10^ ± 0.5 × 10^−10 bc^	0.084 ± 0.02 ^b^
2	2.26 × 10^−10^ ± 0.5 × 10^−10 c^	0.108 ± 0.02 ^b^
3	2.20 × 10^−10^ ± 0.8 × 10^−10 c^	0.105 ± 0.04 ^b^
4	4.57 × 10^−10^ ± 0. 4 × 10^−10 a^	0.221 ± 0.02 ^a^
5	3.05 × 10^−10^ ± 0.3 × 10^−10 b^	0.098 ± 0.01 ^b^
5%CPE	2.96 × 10^−10^ ± 5.83 × 10^−11 b^	0.100 ± 0.016 ^b^
10%CPE	4.69 × 10^−10^ ± 1.25 × 10^−10 a^	0.155 ± 0.047 ^a^
15%CPE	4.13 × 10^−10^ ± 1.19 × 10^−10 a^	0.154 ± 0.045 ^a^

Data are expressed as mean ± standard deviation. Values within the same column followed by the same superscript letter are not significantly different (*p* > 0.05), while different letters indicate statistically significant differences.

**Table 4:** Antioxidant activity of extract-loaded nanoemulsions.

Sample No.	DPPH	Reducing Power	ABTS
	µmol TE/100 mL		
Blank	Na *		
5%CPE	0.04 ± 0.00 ^c^	0.59 ± 0.01 ^c^	1.24 ± 0.01 ^c^
10%CPE	0.07 ± 0.00 ^b^	0.73 ± 0.03 ^b^	1.85 ± 0.01 ^b^
15%CPE	0.19 ± 0.01 ^a^	1.06 ± 0.01 ^a^	2.69 ± 0.05 ^a^

* Negligible activity. Different superscript letters within the same column indicate statistically significant differences between samples (*p* < 0.05).

## Data Availability

The original contributions presented in the study are included in the article/Supplementary Material, further inquiries can be directed to the corresponding author.

## Supplementary Materials

The following supporting information can be downloaded at: https://www.mdpi.com/article/10.3390/foods15111909/s1. Table S1: Droplet size, PDI, and zeta potential values for unloaded nanoemulsions during the monitoring period; Table S2: Droplet size, PDI, and zeta potential values for nanoemulsions prepared with extract concentration variation; Figure S1: Droplet size of loaded nanoemulsions during 14 days of storage.
