# Supplementary material for: Innovative Use of Kiwano Cloud Point Extract in Bioactive Nanoemulsion Development
Source: Foods. 2026 May 28;15(11):1909. doi: 10.3390/foods15111909 (PMC13256982; doi:10.3390/foods15111909)
Supplement: Supplementary file 1 [file foods-15-01909-s001.zip › foods-4173427-supplementary.pdf]

Table S1. Droplet size, PDI, and zeta potential values for unloaded nanoemulsions during the monitoring period.

**Sample**	Droplet size by days (nm)							PDI by days							Zeta Potential (mV) by days						
	1	7	14	21	30	45	60	1	7	14	21	30	45	60	1	7	14	21	30	45	60
1	286.3 ± 6.03	402.2 ± 11.54	277.9 ± 2.34	451.0 ± 14.77	391.5 ± 8.32	313.4 ± 7.88	422.0 ± 10.33	0.58 ± 0.03	0.38 ± 0.05	0.58 ± 0.002	0.65 ± 0.05	0.36 ± 0.06	0.57 ± 0.06	0.60 ± 0.06	-11.93 ± 1.46	-22.37 ± 5.49	-9.71 ± 2.59	-10.34 ± 2.4	-23.87 ± 4.37	-9.04 ± 0.86	-18.8 ± 1.66
2	360.7 ± 1.27	395.8 ± 15.27	523.4 ± 9.87	329.3 ± 25.09	/	/	/	0.40 ± 0.03	0.33 ± 0.01	0.56 ± 0.02	0.65 ± 0.08	/	/	/	-6.18 ± 0.17	-6.33 ± 0.84	-9.49 ± 0.9	-7.73 ± 0.27	/	/	/
3	301.0 ± 12.84	283.4 ± 4.45	381.7 ± 4.23	335.1 ± 4.56	/	/	/	0.62 ± 0.05	0.56 ± 0.01	0.53 ± 0.01	0.49 ± 0.01	/	/	/	-7.46 ± 0.37	-6.75 ± 0.35	-12.37 ± 2.37	-13.57 ± 1.94	/	/	/
4	257.2 ± 16.48	456.6 ± 30.78	443.3 ± 24.99	436.5 ± 26.54	/	/	/	0.55 ± 0.17	0.79 ± 0.23	0.64 ± 0.12	0.57 ± 0.11	/	/	/	-11.60 ± 0.1	-16.06 ± 1.45	-16.53 ± 0.25	-14.8 ± 1.39	/	/	/
5	218.2 ± 5.82	200.1 ± 5.95	329.7 ± 9.11	213.7 ± 1.12	120.9 ± 4.1	141.6 ± 3.15	164.0 ± 5.13	0.49 ± 0.04	0.85 ± 0.05	0.59 ± 0.03	0.49 ± 0.01	0.56 ± 0.04	0.52 ± 0.03	0.58 ± 0.16	-18.90 ± 0.17	-13.80 ± 0.17	-20.2 ± 0.35	-20.37 ± 0.32	-24.70 ± 2.85	-16.43 ± 0.55	-13.63 ± 1.93

Table S2. Droplet size, PDI, and zeta potential values for nanoemulsions prepared with extract concentration variation.

Sample	Droplet size	PDI	Zeta Potential (mV)
5%CPE	168.70 ± 0.44	0.30 ± 0.04	-21.03 ± 0.21
10%CPE	106.74 ± 7.96	0.40 ± 0.07	-14.37 ± 1.21
15%CPE	205.27 ± 22.18	0.37 ± 0.05	-17.00 ± 1.11

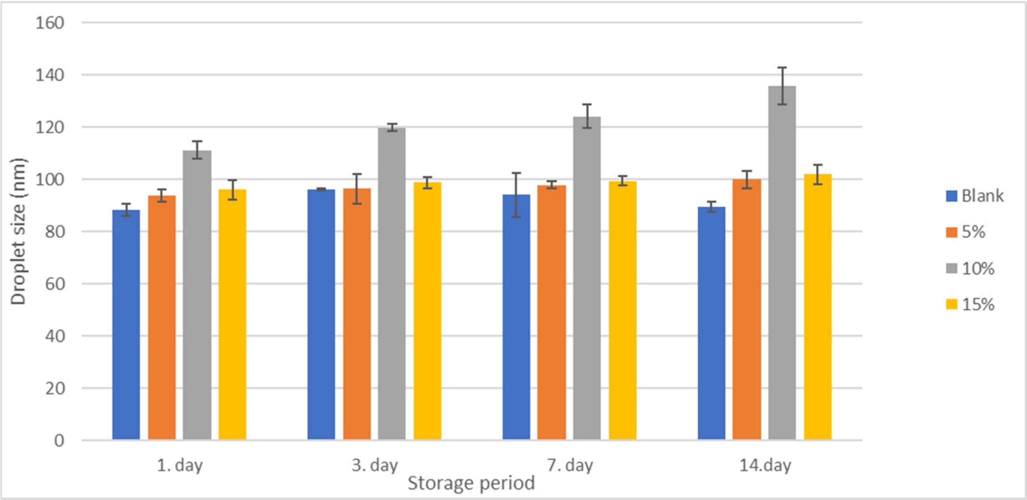

Figure S1. Droplet size of loaded nanoemulsions during 14 days of storage
